# Evaluation of wave configurations in corrugated boards by experimental analysis (EA) and finite element modeling (FEM): the role of the micro-wave in packaging design

**DOI:** 10.1007/s00170-023-11397-y

**Published:** 2023-04-25

**Authors:** Franco Maria Di Russo, Maria Maria Desole, Annamaria Gisario, Massimiliano Barletta

**Affiliations:** 1 Dipartimento Di Ingegneria Meccanica e Aerospaziale, Sapienza Università Di Roma, Via Eudossiana 18, 00184 Rome, Italy; 2 Dipartimento Di Ingegneria Industriale, Elettronica e Meccanica, Università Degli Studi Roma Tre, Via Della Vasca Navale 79, 00146 Rome, Italy

**Keywords:** Corrugated board, Product design, Mechanical properties, Finite element modeling

## Abstract

The aim of this paper is to study the mechanical behavior of corrugated board boxes, focusing attention on the strength that the boxes are able to offer in compression under stacking conditions. A preliminary design of the corrugated cardboard structures starting from the definition of each individual layer, namely the outer liners and the innermost flute, was carried out. For this purpose, three distinct types of corrugated board structures that include flutes with different characteristics, namely the high wave (C), the medium wave (B), and even the micro-wave (E), were comparatively evaluated. More specifically, the comparison is able to show the potential of the micro-wave which would eventually allow a significant saving of cellulose in the fabrication process of the boxes, thus reducing the manufacturing costs and causing a lower environmental footprint. First, experimental tests were carried out to determine the mechanical properties of the different layers of the corrugated board structures. Tensile tests were performed on samples extracted from the paper reels used as base material for the manufacturing of the liners and flutes. Instead, the edge crush test (ECT) and box compression test (BCT) were directly performed on the corrugated cardboard structures. Secondly, a parametric finite element (FE) model to allow, on a comparative basis, the study of the mechanical response of the three different types of corrugated cardboard structures was developed. Lastly, a comparison between the available experimental results and the outputs of the FE model was carried out, with the same model being also adapted to evaluate additional structures where the E micro-wave was usefully combined with the B or C wave in a double-wave configuration.

## Introduction

The corrugated packaging market was valued at $ 172.61 billion in 2021. It is expected to reach $ 212.32 billion by 2027, with a compound annual growth rate (CAGR) by 3.66% from 2022 to 2027 (*Corrugated Board Packaging Market—Growth, Trends, COVID-19 Impact, and Forecasts (2022—2027)*) [14]. Corrugated cardboard packaging is a versatile and economical method to protect, store, and transport various products, and its main characteristics have made it an integral component of the packaging industry. Corrugated cardboard, in fact, allows easy conditions of transport and storage of the products, favored by the low weight and high strength of the material, as well as by its economic convenience [41]. Another aspect that makes corrugated cardboard a widely used material in the packaging industry is inherent in its excellent environmental performance, in particular it is totally recyclable, and being biodegradable it can also be composted [15]. The culture of good governance to address sustainability issues in an increasingly stringent manner has encouraged a large increase in their employment in recent years. In [32], recyclable corrugated cardboard boxes were assessed as more sustainable than high-density polyethylene boxes. Indeed, the e-commerce industry has provided a decisive boost to the corrugated boxes market. In fact, leading e-commerce companies (such as Amazon.com, Inc.) have extensively used corrugated boxes as their primary packaging. As a result of the COVID-19 pandemic, the demand for products sold on e-commerce platforms has increased drastically, therefore also that of packaging. Corrugated board, in this sense, represents an effective solution, for example, for the packaging of cosmetics, hi-tech products, clothing, and other kinds, as it keeps away moisture and resists to longer shipping times. These aspects are relevant for the customer satisfaction, who need products to be delivered in a short time and received in good order. Generally, corrugated cardboard packaging has three layers in its mono-wave configuration, the flute is the layer that presents the corrugation, while the liners, the outermost layers, are those that provide resistance to bending [51]. Liners and flutes are usually glued along the edges to form a sandwich panel. Corrugated cardboard is an orthotropic material,consequently, the mechanical characteristics vary along the three directions. The anisotropic, in this case orthotropic, behavior of the material is due to the fiber orientation process during the manufacturing process. The directions to be studied are essentially three: machine direction (MD, transverse direction (CD, and direction along thickness (TD [2]. The elastic modulus varies according to the direction but also according to the grammage. Normally, with the same type of paper, the elastic modulus increases as the grammage increases. However, the production conditions, such as the production speed, can affect the orientation of the fibers and therefore the mechanical properties [13]. As for the direction, the elastic modulus is greater along the MD rather than in CD. Lastly, smaller values are to be expected in the TD. This is always considered valid, both under standard conditions (23 °C and 0% RH) and refrigerated conditions (0 °C and 90% RH) [16]. Several researchers have analyzed the mechanical behavior of corrugated cardboard using different approaches: analytical, experimental, and numerical [9, 18, 20, 25, 41, 50]. There are also different approaches when it comes to the evaluation of the specific properties to test for corrugated cardboard structures, such as resistance to bending, crushing, compression, sliding, instability, or final failure [17].

[33] have examined the non-linear mechanical response of corrugated cardboard through experimental bending tests from an analytical point of view, as well as [24] which also considers the edge crush test (ECT) and used experimental data as input to model different load geometries with finite elements. [10] studied the instability behavior of corrugated paper packaging from a theoretical–experimental point of view, setting a finite element modeling with which it was possible to reproduce the instability loads obtained from the ECT test and study the compressive strength of the box, by box compression test (BCT) analysis. Also [41] set up an experimental numerical analysis aimed at predicting the breaking load for corrugated cardboard boxes. By analyzing two “standard” wave configurations, medium (type-B) and high (type-C), they found simulated values, respectively, 3% higher and 5% lower than the values extracted from the experimental campaign. [9] developed an FE model to analyze the instability and post-instability behavior of a corrugated cardboard box in terms of critical loads and load–displacement curves using boxes with different geometries and materials to show their effect on stiffness to compression. He found the material can be considered as an elastic linear orthotropic material [9]. Other authors analyzed the behavior of cardboard packaging subjected to static compressive loads, through a non-linear finite element analysis [27, 54] based on large displacements and plasticity [8]. In particular, the small size of the corrugated cardboard grooves requires the use of detailed 3D numerical models of the entire structure requiring excessive calculation time [36, 50, 54]. For this purpose, to characterize the mechanical properties on a macro-mechanical scale, the homogenization process is used, which allows to transform the corrugated cardboard into an equivalent homogeneous plate [17]. This approach allows to speed up the analysis and at the same time provide accurate answers [12, 49]. Biancolini et al. [11] highlighted the consistency of the homogenization process through the development of two numerical finite element models: a FE model made with homogenized elements and a FE model that represents the entire geometry of the corrugation. The numerical results of the ability to withstand stacking loads obtained with both FE models were consistent with the experimental results. The homogenization process is reported in numerous studies in the literature [1, 22, 28, 34, 49] since it is fundamental in the analysis of box compression behavior, given its orthotropic material nature with corrugation. For the implementation of the constitutive model of the corrugated cardboards, the linear problem can be studied through Hooke’s law for orthotropic materials, while the plastic behavior is regulated by the quadratic criterion of Hill yield [47]. In [26], the theory of laminates is applied for the implementation of the analytical model, considering a corrugated cardboard made of several sheets of orthotropic paper, of uniform thickness, so that each layer does not move with respect to the others. In order to determine the analytical model of the cardboard, a point-type lamination technique can also be used, through laminate theory [2, 30, 48]. The analysis adopted is based on finite element modeling, already carried out in other studies [17, 19].

More recently, the growing concerns for environmental impacts of packaging solutions and the continuous efforts to reduce the shipment cost are drawing worldwide an increasing attention of the academics, technicians, and practitioners to innovate the design of corrugated boxes. Basically, the weight, the type of wave configurations involved, and the geometry of the boxes must be highly customized to meet only the most stringent needs of packaged items. In a recent study, [39, 40] developed a numerical and computational analysis with the aim of determining the influence of grammage on the mechanical properties of corrugated cardboard. The study assesses the sensitivity of the edge crushing resistance, the critical load, and the box strength to static crushing resistance when the grammage of the individual layers of the corrugated cardboard change. This effort is paid to support a more conscious choice of the composition of multi-layer corrugated boards. For this purpose, single-wave configurations from A to E and possible multi-wave pairs are analyzed: BC, BE, AE, FE, or EB. In another recent study [45], an experimental–numerical approach was followed to measure the compressive strength of the box in double-wave configurations. Specifically, the study considers the F micro-wave paired with bigger waves in two single-wall (A/F and B/F) and two double-wall (BB/F and AB/F) configurations. To date, despite a growing interest in the optimization of corrugated carboard design, a very limited number of studies has focused on the use of single-wave cartons using an E-type micro-wave structure. Usually, the latter is coupled with a medium or large wave to make a double-wave cardboard. However, cardboard boxes with micro-waves alone would have a great potential in shipping of electronic products, clothing, or various items of a low weight. The adoption of the micro-wave alone would allow to obtain light and easy to handle packaging, as well as a reduction in the amount of material used for packaging. In addition, the usage of the micro-wave would reduce the impact on the total cost of the shipment itself, also reducing the related environmental impact.

This is, therefore, the context in which the present paper aims to study on a comparative base the mechanical behavior of corrugated board boxes, focusing attention on the strength that the same boxes are able to offer in compression under stacking conditions by changing their structure (i.e., waves configuration). In the first instance, the design of the corrugated cardboard structures is comparatively evaluated changing the wave geometry (i.e., standard, double-, and micro-wave was studied). Additional geometrical configurations achieved by pairing the micro-wave (type-E) with the broader type-B or C waves were also investigated to assess their potential in packaging of heavier goods. The mechanical properties of the base papers used for the design of the corrugated board structure and the resulting structures were also examined. A finite element model was built for the evaluation of the mechanical response of the different corrugated cardboard structures. Finally, the reliability of the model was evaluated by comparison with the experimental results.

## Materials and methods

### Materials

For the analysis of the corrugated cardboard box, three different single-wave scenarios were considered. In particular, each scenario consists of a set of cards specifically chosen to evaluate variations in terms of mechanical-structural behavior. In the first and second scenario, the same type of paper was chosen for the liners, namely a Test liner paper with a weight of 135 g/m^2^. In the first scenario, the wave referred to was a high wave, type C, made with paper Use semichemical with grammage 115 g/m^2^, while in the second the wave referred to was a medium wave, type B, made with paper Semichemical use with grammage 140 g/m^2.^

The third scenario involved the use of a micro-wave E of the Medium type with a grammage of 120 g/m^2^ for the flute, while Test (white in color and named Tb) for the outer and Liner for the inner, with weights of 125 g/m^2^ and 120 g/m^2^ respectively. The three scenarios are respectively identified by the following abbreviations: TUT/323/C, TUT/363/B, and TbML/242/E. All cards referred to are recycled papers. In Table 1, the main geometric characteristics for each type of wave considered are then specified.

**Table 1:** Geometrical characteristics of the waves analyzed

Wave profile	Wave height (mm)	Wave coefficient	Wave pitch (mm)	N° of wave per meter
C	4.2	1.44	7.6	124
B	2.6	1.36	6.25	155
E	1.2	1.25	3.3	296

### Methodology for mechanical characterization

#### Tensile test

**Fig. 1:**
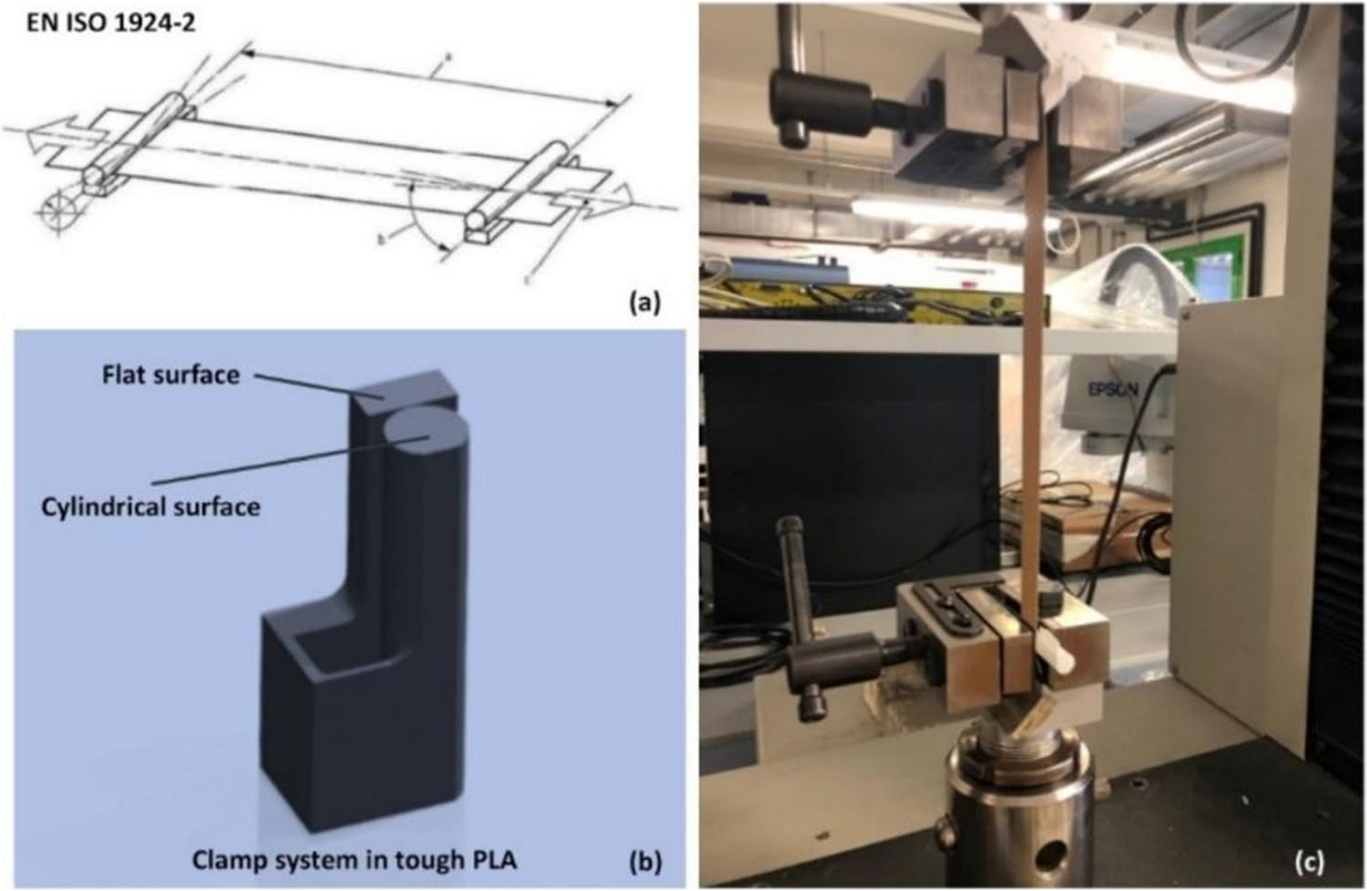
**a** Technical requirements for the clamping system based on EN ISO 1924–2:2008, **b** 3D model for clamping system. It can grip the test piece between a cylindrical and a flat surface firmly, without damage or slippage; **c** specimen before tensile test

In order to define the mechanical parameters of the papers, tensile tests were preliminarily carried out on paper specimens with dimensions of 200 ± 1 mm × 15 ± 0.1 mm. The thickness of the specimens was measured with a Palmer micrometer, making 5 measurements over the entire length of the specimen, and making an average of these measurements. Table 2 shows the values of the measured average thickness. The standard followed for carrying out the tensile tests is UNI EN ISO 1924–2-2008, which specifies a method for measuring the tensile strength, deformation at break, and the absorption of tensile energy of paper and cardboard. To ensure a correct grip of the sample, avoiding the concentration of tensions, special supports in technical PLA were manufactured using the Ultimaker Model S5 FDM 3D printer (Fig. 1) and then inserted into the grips of the traction machine. The machine used for the tests is the MTS, Insight 5 model, equipped with a 2.5-kN load cell. In order to ensure a good degree of reliability of the results, 10 replications were carried out for each card. As previously stated, paper is an orthotropic material, for these reasons the elastic modulus must be measured along the three directions MD, CD, and TD (*i.e., x, y, and z respectively)*.

**Table 2:** Indication of measured average thickness

Scenario	Paper type	(mm)
1		
Inner and outer	TL135	0.2
Flute	US140	0.21
2		
Inner and outer	TL135	0.2
Flute	US115	0.16
3		
Inner	L120	0.19
Outer	TB125	0.15
Flute	M120	0.17

1 $$K= \left[\begin{array}{cccccc}\frac{1}{{E}_{MD}}& \frac{{-\nu }_{xy}}{{E}_{CD}}& \frac{{-\nu }_{xz}}{{E}_{ZD}}& 0& 0& 0\\ \frac{{-\nu }_{xy}}{{E}_{MD}}& \frac{1}{{E}_{CD}}& \frac{{-\nu }_{yz}}{{E}_{ZD}}& 0& 0& 0\\ \frac{{-\nu }_{xz}}{{E}_{MD}}& \frac{{-\nu }_{yz}}{{E}_{CD}}& \frac{1}{{E}_{ZD}}& 0& 0& 0\\ 0& 0& 0& \frac{1}{{G}_{yz}}& 0& 0\\ 0& 0& 0& 0& \frac{1}{{G}_{xz}}& 0\\ 0& 0& 0& 0& 0& \frac{1}{{G}_{xy}}\end{array}\right]$$

Through the tensile tests, it was possible to derive the elastic properties in the plane, therefore along MD and CD, while in the direction of the thickness the elastic modulus can be obtained through the following expression [35]:

2 $${E}_{ZD}= \frac{{E}_{MD}}{200}$$

*E*_*MD*_ is the elastic modulus along the machine direction, while *E*_*ZD*_ is the elastic modulus along the thickness. The elastic modules were obtained through the formulas defined by [7].

3 $${G}_{xy}=0.387\sqrt{{E}_{MD}{E}_{CD}}$$

4 $${G}_{xz}= \frac{{E}_{MD}}{55}$$

5 $${G}_{xz}= \frac{{E}_{CD}}{55}$$

where *E*_*CD*_ is the elastic modulus along the transverse direction.

For the liners, Poisson’s ratio was ${\nu }_{xy,\mathrm{liner}}=0.34$, while for the flute was ${\nu }_{xy,\mathrm{flute}}=0.33$, as specified by [10]. The coefficients ${\nu }_{yz}$ and ${\nu }_{xz}$, for both cases, were determined through [41] and were considered equal to 0.01.

#### ECT

**Fig. 2:**
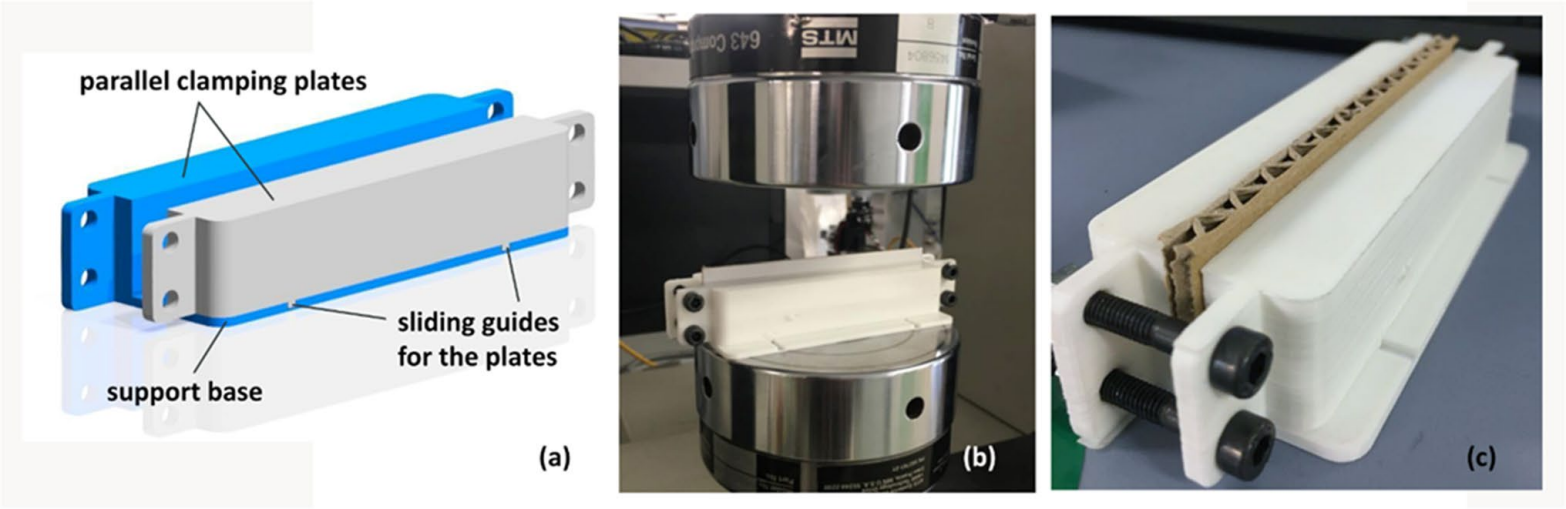
**a** Clamping system for the ECT specimen, **b** image of TUT/363/B specimen fixed in clamping system and before the ECT test, **c** image of the TUT/363/B specimen after the ECT test

The best predictor of the compressive strength of corrugated cardboard boxes is the ECT test value [45]. The purpose of the ECT test is to identify the maximum load that the corrugated cardboard structure can withstand before it fails. Corrugated cardboard strength measurement is the dominant factor that predicts vertical stacking performance of boxes [38] and the durability of boxes [4]. In the present study, the ECT test was conducted in order to evaluate the behavior of the three different configurations chosen: TUT/323/C, TUT/363/B, and TbML/242/E in case of stacking. The standard referred to is ISO 3037. The standard procedure requires the application of a compression load on a specimen of fixed dimensions, 100 mm long and 25 mm wide, bound by a joint on the lower surface and on the lateral surfaces up to 5 mm from the upper end. Each specimen was then positioned inside a special gripping system and supported along the edge between two parallel plates that can slide along special guides and move closer to ensure contact with the external faces of the specimen, the two liners. The gripping system was made in technical PLA using FDM 3D printing technology (Ultimaker S5 model) and is shown in Fig. 2. The load direction to be applied is parallel to the grooves, i.e., the transverse direction (CD) of the cardboard so as to reproduce the load direction of the corrugated cardboard boxes when assembled. The machine used is the MTS Insight 5, equipped with a 2.5-kN load cell. Also in this case, for each configuration of the structure under examination, 5 replications were carried out. The test speed, according to regulations, has been set at 20 mm/min.

#### BCT

**Fig. 3:**
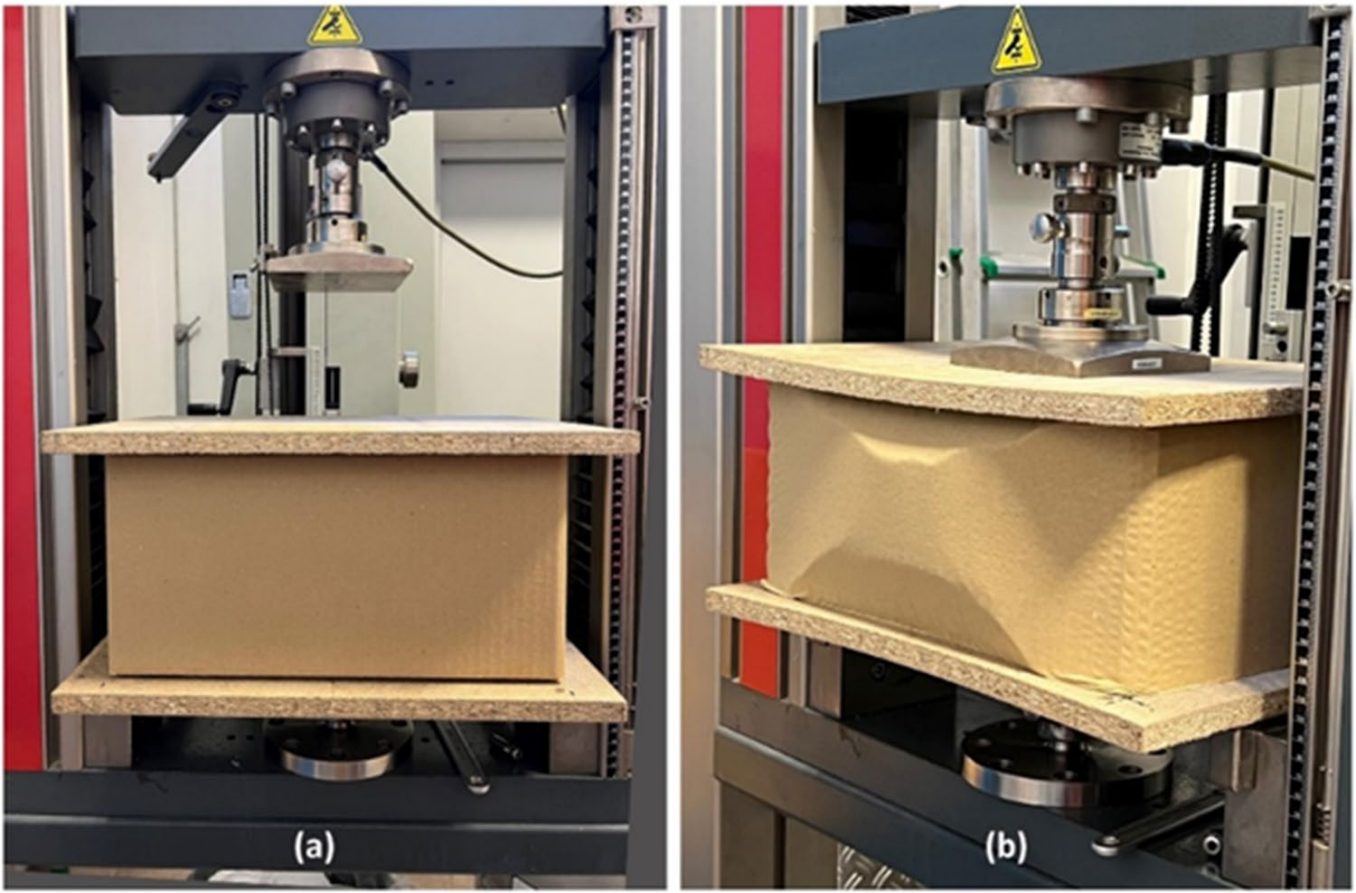
**a** View of the placement of the American box (scenario 1) between two parallel planes before BCT test; **b** deformation of American box after BCT test

The box compression test is a standardized test that quantifies the compression strength of the packaging containers and ensures stronger and better-quality packaging containers. The BCT is the most commonly used test to obtain data on the load capacity of a box, and it principally allows to measure the resistance to vertical compression, i.e., how many kilograms the box can withstand before deforming. The BCT was conducted on empty boxes measuring 330 × 265 × 150 mm^3^ made in the three configurations before described. The machine used for these tests is the Zwick-Roell, equipped with a 10-kN load cell. The standard referred to is the FEFCO TM 50 or DIN 55,440–1 regulation, in which the test speed is prescribed at 13 mm/min. For each configuration of the structure under examination, 5 replications were carried out as usual. A correct experimental set-up system requires the use of two parallel rigid plates to evenly distribute the compression load. So, the lower plate is fixed to the base of the plate, while the upper plate is placed in contact with the upper surface of American box (Fig. 3). However, to verify that the plates were parallel, a bubble level was used and the distances between the corners of the rigid plates were measured with a digital vernier caliper, as proposed by [37].

#### ECT and BCT correlation through McKee’s formula

McKee et al [38] provided a correlation between the ultimate compressive load of a corrugated box and the load at which buckling occurs. In fact, through compression tests on boxes, it was shown that after reaching buckling of the structure, most of the compressive strength is concentrated near the edges without reaching collapse. On the other hand, the edges are geometrically more stable areas. The proposed method was able to relate compressive strength to ECT, paper bending stiffness, and box size. This method uses the basic parameters of corrugated board and some empirically determined correction factors that increase the accuracy of the results obtained. However, it should be noted that the use of correction factors may reduce the universality of the method, especially since the values must be determined individually. In practice, different corrugated board types and paper quality levels must be considered for each specific packaging project.

The basis for the derivation is the empirical formula for the breaking load *P*_*f*_ [N/mm], which according to the authors is the result of a combination of compressive strength and peak load, namely:

6 $$\frac{{P}_{f}}{{P}_{cr}}=k{\left(\frac{\mathrm{ECT}}{{P}_{cr}} \right)}^{r}$$

where *k* and *r*, with *r* ϵ (0, 1) (most often *r* = 3/4), are two empirical constants; ECT is the compressive strength of the corrugated board, expressed in newtons per millimeter; and *P*_*cr*_ is the critical load value, expressed in newtons per millimeter. The latter is derived from the phenomenon of buckling of the vertical walls of the box. The calculation of ultimate load using McKee’s formulation is particularly time-consuming and complicated to implement in the industrial field. For this reason, a “simplified” formulation is suggested. In the present study, the simplified formulation was used. The formula was referred to the types of packaging defined as “American,” that is, characterized by a height-to-perimeter ratio of about 1.5–1.7. Boxes should have no knurling or perforated handles that could alter the seal. This formula is as follows:

7 $${P}_{\mathrm{box}} = {k}^{-\gamma }\mathrm{ECT} \sqrt{t} \sqrt{{p}_{b}}$$

where

*t*: thickness of the corrugated cardboard
*p*_*b*_: box perimeter

The value of the constant *k*, which is 1.82, is dependent on the units in which the other quantities are expressed. The factor *g* takes into account the geometric characteristics of the box and allows for more consistent results. It has been set at 0.333. The value of ECT must be expressed in kilonewtons per meter. The perimeter and thickness, on the other hand, in centimeters and millimeters, respectively. The load value obtained as a result of the formula will then be expressed in kilograms.

## FE model

To support the experimental activities, a parametric model was built to simulate the structural and mechanical behavior of the three configurations chosen for the analysis, distinguished by type of paper and wave. The finite element model was implemented using the commercial software “Ansys Workbench 2020 R2,” an engineering simulation software that allows you to solve complex structural engineering problems through finite element analysis. The simulation activity was aimed at predicting the results of the experiments in the ECT and BCT tests described above, as well as illustrating how the different combination of materials affects the load compression curve.

### Generation of geometry and FE model for simulation of the ECT test

**Fig. 4:**
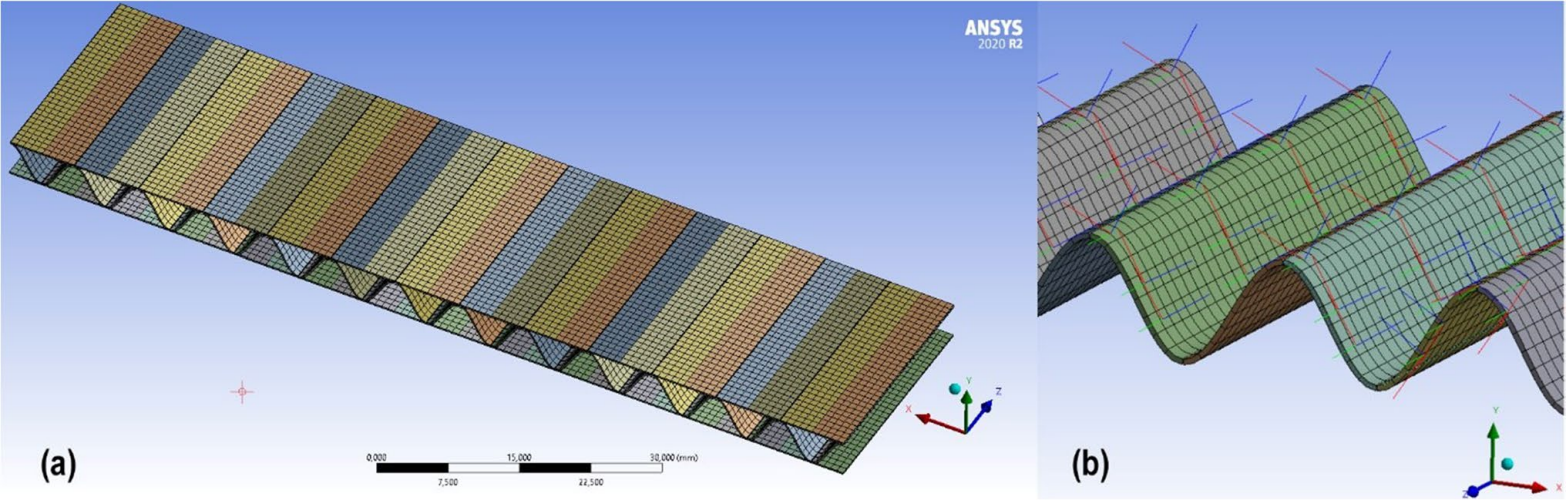
**a** Finite element mesh of the ECT specimen, **b** detail of the local reference system for the flute

The 3D model imported into ANSYS was generated using commercial CAD software “Autodesk Inventor Professional 2022,” pursuing a parametric modeling logic. The size of the ECT specimen is to be considered analogous to that of the actual specimen used in the experimental tests, so that a subsequent comparison between experimental and numerical results can be made. The standard referred to is ISO 3037, which suggests sample size ECT corresponding to 100 × 25 mm. The model was created assuming that the flute geometry can be approximated by a sine wave [11]. To model the entire geometry, however, reference was made to a periodic structure, consisting of a flute section and two liners of length corresponding to the flute pitch. The number of occurrences was then determined, in order to build a model of 100 mm in length. As regards the relative contact areas between the liners and the flute, it has been assumed that the three elements constituting the corrugated cardboard are glued together. This condition was managed by imposing a share between the nodes of the elements at the interface and the imposition of a *Bonded* contact between the elements in contact, preventing the separation between faces or edges and sliding [24, 27]. Therefore, working in this way, the discretization elements used in the mesh and constituting the two contact surfaces find themselves sharing the same nodes, in correspondence with the contact area. The element chosen for the discretization of the geometry is the shell with a quadratic function [50, 54]. Figure 4a shows the generation of the mesh on the ECT specimen. Corrugated cardboard is a strongly anisotropic material, for these reasons the mechanical behavior of the materials constituting the configurations under examination has been approximated as an elastic linear orthotropic [17]. By definition of orthotropic mechanical properties, it was necessary to correctly orient the material properties by defining a multitude of local reference thresholds (Fig. 4b) taking into account the geometry of the elements constituting the structure under examination. This is mainly necessary for an appropriate orientation of the material properties along the flute.

**Fig. 5:**
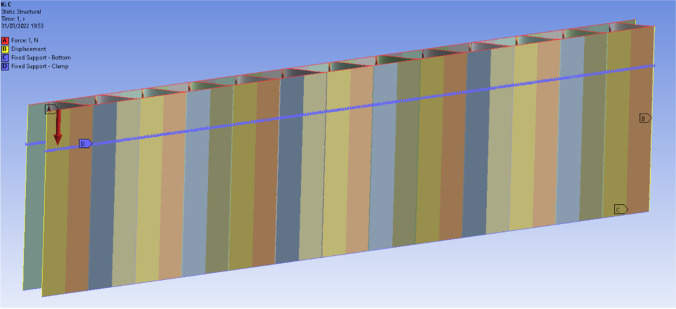
Boundary conditions of the ECT specimen

Furthermore, in order for the edge crush test to be reproduced in a realistic and reliable way, attention had to be paid to the setting of the simulation boundary conditions (Fig. 5). The constraint arrangement chosen involved the imposition of an interlocking constraint at the lower edges of the specimen. Further *fixed constraint* was imposed on the nodes belonging to the two outer liners at the value of ordinate *z* = 20 mm. An intensity load of 1 N was then applied to the upper edges of the specimen. The choice of the intensity load applied is related to the eigenvalue buckling analysis, although it is possible to evaluate the value of the *load multiplier* that, multiplied by the applied intensity load, provides the value of the critical buckling load.

### Mesh convergence analysis

A study of mesh convergence was carried out in order to obtain precise results by balancing the size and resolution time of a model. The study of mesh convergence is fundamental and in scientific literature some techniques are described to evaluate the convergence errors of the mesh [42, 46]. Such errors in convergence make it possible to define the accuracy of the numerical solution, despite not knowing the exact solution [3].

#### Estimation of mesh discretization error

Displacement is the unknown variable in finite element analysis. It is calculated in each node of the model. The stresses are obtained from the first derivative of the displacement field and are calculated using the equation:

8 $$\left[F\right]= \left[K\right]\left[n\right]$$

with [*K*] reference is made to the global stiffness matrix, while with [*n*] and [*F*] to the displacement and force vector respectively. The stress values are an average of the stresses of all the elements connected to the node and this introduces the magnitude error of the stress value; the coarser the mesh, the greater the difference between the stress values of the adjacent elements [3]. As the mesh density increases, the nodal and elemental stress values are more similar, and the accuracy also increases and the time required for simulation also increases [46]. In particular, in this case study, the use of shell elements led to a thorough verification, in the aspect ratio. The thickness of the shell element must be “thin” compared to the planar dimensions of the element. To set the same mesh criterion for each structure under consideration, a relationship between the wave pitch and the mesh pitch size was assumed, the latter corresponding to a tenth of the flute pitch corresponding to 0.76 mm, 0.625 mm, and 0.55 mm for C, B, and E wave respectively. However, the E wave, being a micro-wave, represented a special case; therefore in order to avoid incurring a ratio of wrong shape, with thickness of the not “thin” quadratic elements, the dimension of the mesh step has been increased.

Also for the outer and inner, it was decided to choose the same dimension of the mesh pitch of the studied wave, after a procedure of gradual thickening of the mesh. This choice was supported by the fact that the increase in solution times was not excessive. The mesh showed a good degree of homogeneity, also there was a good discretization at the contact zones and the distortion of the elements was contained. Verification parameters used to identify the distortions were essentially three: element quality, aspect ratio, and skewness [52]. The first parameter allows to evaluate the quality of the mesh and avoid inaccurate and incomplete solutions. The average value which was found for the parameter “element quality” is 0.9991, thus indicating a correct discretization activity. The shell elements used are perfect squares. For the second parameter, the aspect ratio, the resulting average value is 1.0197. Most of the elements are extremely close to the unit value; therefore, all the shell elements have an adequate shape ratio, matching the condition of “thin thickness.” Skewness average value is 0.0012 confirming a correct setup of the mesh conditions. The discretization elements are all equilateral, so the mesh which is smooth and homogeneous. The trend is also confirmed for wave B and wave E. On the basis of the previous estimates, the mesh discretization thus set counts a number of elements and nodes which is a function of the type of wave. Table 3 shows a detail of the choices made on the mesh for the types of waves under examination.

**Table 3:** Edge crush test — data mesh

Wave	Shell elements		
	Dimension (mm)	Nodes (number)	Elements (number)
Single wave			
C	0.76	15,444	15,444
B	0.625	63,938	21,760
E	0.555	27,000	27,000
Double wave			
BE	0.625	110,804	39,003
CE	0.625	110,804	39,003

### Generation of geometry and FE model for simulation of the BCT test

**Fig. 6:**
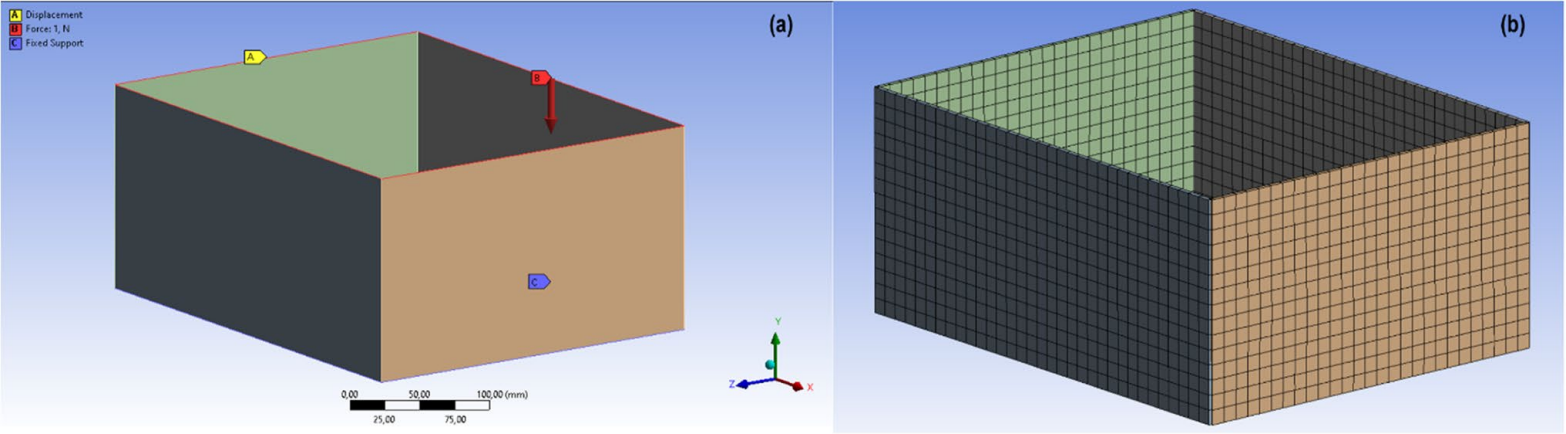
**a** 3D model of the American box with constraint conditions; **b** 3D model discretization

The CAD model was built respecting the geometry and dimensions corresponding to the geometry of the American boxes tested experimentally (Fig. 6a). The top and bottom closure flaps of the packaging were not considered. This is because these are elements that are not directly affected by the surrounding conditions of the problem, so their presence, or absence, is to be considered negligible [10]. In order for the corrugated box model to reproduce the actual physical behavior, it is necessary to pay attention in the definition of the boundary conditions of the problem. Two boundary conditions were imposed: *fixed support* at the lower perimeter of the box and a load evenly distributed on the upper perimeter of the box, with an intensity of 1N. Finally, a non-linear resolution procedure of the large displacement type is set up, in order to take into account the non-linear geometry characterizing the corrugated structures [17]. For the discretization of the geometry, also in this case, it has been proceeded through the approach described above for the FEM ECT model. The discretization of the mesh, which followed the evaluation criteria previously described, takes place through the adoption of quadratic shell elements, with element size fixed at 20 mm (Fig. 6b). The mesh, thus set, counts 9303 elements and 9574 nodes.

### Eigenvalue instability analysis

Eigenvalue instability analysis is commonly used to predict the critical buckling loads of stiff. Stiff structures are designed to withstand loads essentially by axial or in-plane action, rather than by bending action. Typically, very small deformations take place in those structures before buckling can occur. However, even when the response of a structure is non-linear before collapsing, a general analysis of the buckling of the eigenvalues can provide extremely useful insights into how the structure will collapse. Buckling loads are calculated according to the original status of the structure. The equation applied to the instability analysis is as follows:

9 $$\left(\left[K \right]- \lambda i \left[P\right]\right)\left\{\varphi i \right\}= 0$$

where $\left[K\right]$ is the structural stiffness matrix and $\left[P\right]$ is the preset load matrix while $\left\{{\varphi }_{i}\right\}$ represents the buckling mode performed by the structure. Solving the above equation, it is possible to obtain the minimum eigenvalue $\lambda \mathrm{min}$. So, the critical load of the structure can be written as:

10 $$\left\{{P}_{Cr}\right\}=\lambda \mathrm{min}\left\{P\right\}$$

This analysis was conducted in Ansys Workbench using the ready-to-use tool “eigenvalue buckling.”

#### Non-linear instability analysis

Non-linearity is composed of material non-linearity and geometric non-linearity. The first non-linearity refers to non-linear mechanical properties, such as the elastic modulus; geometric non-linearity, on the other hand, implies a change in the geometry of the structure. To solve these problems, in particular with regard to noncomplex geometric non-linearities, an analytical solution can be obtained in some circumstances. The analysis of non-linear instability can be addressed by solving the equation:

11 $$\left[K \right]\left\{{\varepsilon }_{\mathrm{tot}}\right\}= \left[P\right]$$

where $\left\{{\varepsilon }_{\mathrm{tot}}\right\}$ is the total deformation, $\left[K\right]$ is the structural stiffness matrix, and $\left[P\right]$ is the load matrix preset. To solve the above equation, the critical load is divided into certain steps of load increments, indicated below. For each load application of $\left\{\Delta P\right\}$, the load-strain curve is linear. Linear and non-linear processes thus overlap. The load is applied gradually and reaches a critical value when the slope of the load–displacement curves is close to zero, with a further load increase, when a negative slope is obtained. A static structural analysis shall be performed before or in combination with an analysis of instability.

### Homogenization

Corrugated cardboard is an orthotropic material characterized, in the single-wave configuration, by the presence of three layers, two of which are linear, and one is the corrugated wave. For the constitution of an analytical model, it is necessary to solve the geometric problem related to it; to do this, it is necessary to proceed through a process of homogenization of the structure, replacing the flute with a laminate that has equivalent mechanical characteristics, applying the CLPT theory (Classical Laminated Plate Theory). The analytical homogenization process of the wave has been extensively treated in the literature and some scientific papers are of useful reference, such as [30] concerning the modeling of the elastic behavior of woven composite materials and [2, 5, 28, 49, 50], and [23] regarding corrugated cardboard. Corrugated cardboard is considered as an orthotropic sandwich; once the wave is homogenized, the whole structure is homogenized. The present structure has been homogenized referring to the treatises of [10] and [50] which provide for the application of the thin plates theory of Kirchhoff–Love. To homogenize the flute, a unit cell of corrugated cardboard is considered. More specifically, the structure is considered as a set of infinitesimal elements *dx*, so that for each element it is possible to apply the theory CLPT. ${ABD}_{i}$ represents the stiffness matrix for every single element right infinitesimal. The local stiffness of each element depends on the elastic properties of the element and the orientation of the flute, indicated by the angle of inclination of the corrugated sheet *θ*(*x*). This angle is calculated as a function of the distance from the middle line of the corrugated sheet *H*(*x*) [50]:

12 $$\theta \left(x\right)={\mathrm{tan}}^{-1}\left(\frac{dH\left(x\right)}{dx}\right)$$

13 $$H\left(x\right)=\frac{{h}_{c}}{2}\mathrm{sin}\left(2\pi \frac{x}{{f}_{p}}\right)$$

*h*_*c*_ is the height of the core and *f*_*p*_ is the flute pitch. Having defined the stiffness matrix $\left[Q\right]$, the rigidity coefficients of the plane can be evaluated as follows:

14 $$\left[A\left(x\right)\right]= {\int }_{-\frac{H}{2}}^\frac{H}{2}\left[Q\right] dz={[Q}_{o}]{t}_{o}+{[Q}_{i}]{t}_{i}+{[Q}_{c}]{t}_{vc}$$

15 $$\left[B\left(x\right)\right]= {\int }_{-\frac{H}{2}}^\frac{H}{2}\left[Q\right]z dz={[Q}_{o}]{z}_{o}{t}_{o}+{[Q}_{i}]{{z}_{i}t}_{i}+{[Q}_{c}]{{z}_{c}t}_{vc}$$

16 $$\left[C\left(x\right)\right]= {\int }_{-\frac{H}{2}}^\frac{H}{2}\left[Q\right]{z}^{2} dz={[Q}_{o}]({z}_{o}^{2} {t}_{o}+ \frac{1}{12}{t}_{o}^{3})+{[Q}_{i}]{({z}_{i}^{2}t}_{i}+\frac{1}{12}{t}_{i}^{3})+{[Q}_{c}]\left({{z}_{c}^{2}t}_{vc}+\frac{1}{12}{t}_{vc}^{3}\right)$$

where $t$ and $z$ refer respectively to the thickness of the cardboard layers and their vertical position, instead ${t}_{c}$ and ${t}_{vc }= \frac{{t}_{c}}{\mathrm{cos}\theta }$ are the wave thickness and the wave thickness resulting from a vertical cut. Subscriptions $i$, $o$, and $c$ refer in order to the inner, outer, and corrugated liner. Then, the global matrices ${\left[A, B, D\right]}_{\mathrm{global}}$ of all the infinitesimal *dx* elements are calculated numerically from the local matrices [*A*(*x*)], [*B*(*x*)], and [*D*(*x*)] considering an average value in the “*x*” direction by the integral formula [5]:

17 $$\left[\mathrm{A}\right]=\frac{1}{{f}_{p}}{\int }_{0}^{{\mathrm{f}}_{p}}\left[A\left(x\right)\right]dx$$

18 $$\left[B\right]=\frac{1}{{f}_{p}}{\int }_{0}^{{\mathrm{f}}_{p}}\left[B\left(x\right)\right]dx$$

19 $$\left[D\right]=\frac{1}{{f}_{p}}{\int }_{0}^{{\mathrm{f}}_{p}}\left[D\left(x\right)\right]dx$$

The ${\left[ABD\right]}_{\mathrm{flute}}$ can then be determined, and the stress and strain induced in the structure.

For the homogenization of the whole structure, it is necessary to insert into the input the values of the mechanical parameters calculated using the described above formulas (1, 4) and using the elastic properties determined experimentally for the cards tested, along the 3 directions, and those relating to homogenized flute. To solve the problem, the Matlab SW was used for the development of a Matlab code that allows to provide the numerical solution with adequate precision. At this point, the stiffness matrix *Q* is calculated for each single layer, using the elements of this matrix it is possible to derive the stiffness matrix [*ABD*], which allows to define the elastic properties of the entire structure, and the relationship between the applied loads on the average surface and the deformations and rotations that it undergoes. The submatrix B is the extension-bending coupling matrix and is null having assumed a state of plane-stress. For the determination of the matrix [*ABD*] of dimension 6 × 6, reference is made only to the submatrix *A* of extensional stiffness in the plane and to the submatrix *D*, that is, the submatrix of bending stiffness. A reference system shall be taken into account for the calculation of the elements of sub-matrices *A* and *D*, in which the *z*-position of each layer is identified, and a mean axis is defined at the flute centerline.

## Results and discussion

### Tensile tests

**Fig. 7:**
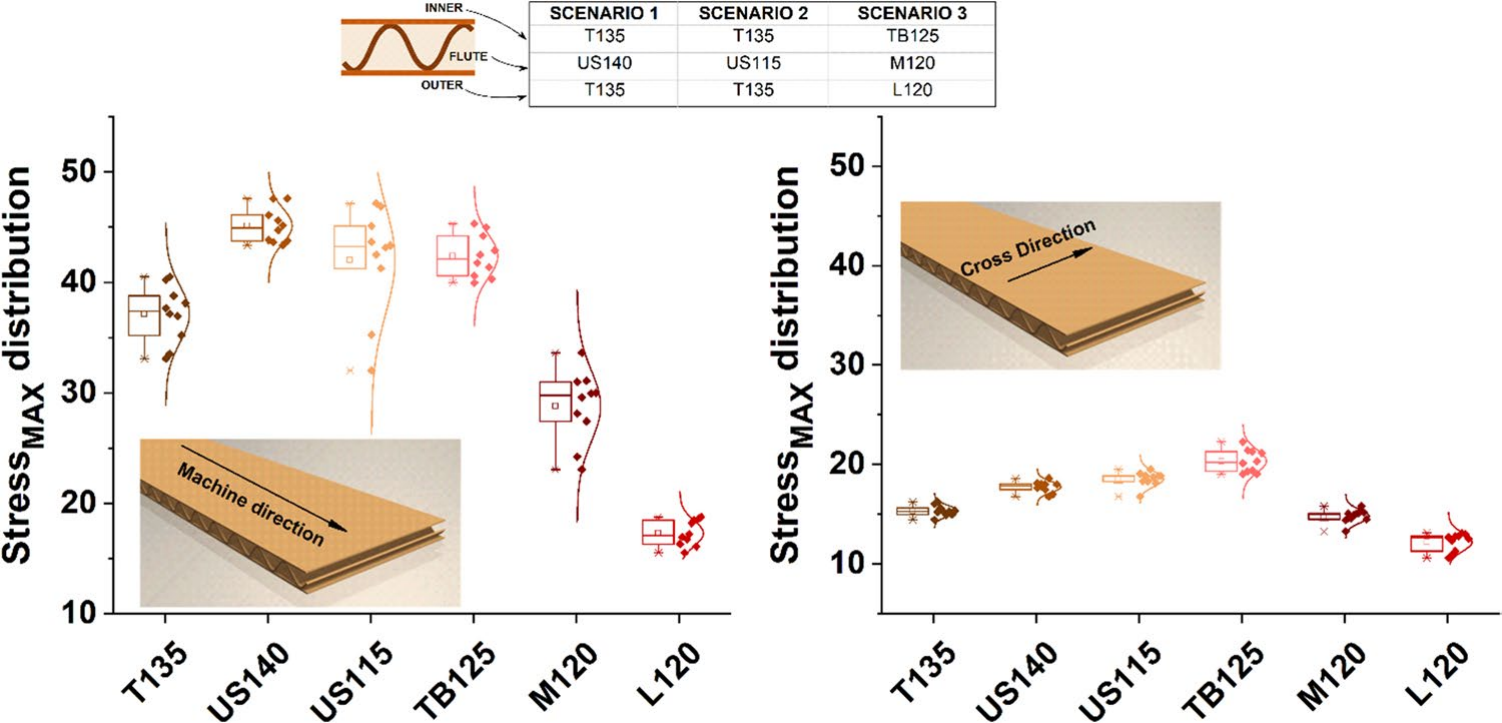
Box chart with normal distribution curve of the paper samples in machine and cross direction

Figure 7 shows the trend of the distribution of the maximum breaking stress evaluated on 10 replicates, for the various samples along the MD direction (left) and CD direction (right). The average values along the MD direction are higher than the CD direction [51]. This is explained by the fact that paper can be considered a fibro-composite material, with a marked anisotropy due to the preferential alignment of the fibers in the direction of the machine as a result of the manufacturing process. Referring to the average value of the maximum stress, it can be seen that for each card it is 40–50% lower in the CD direction, except for the L120 card which shows a less marked difference. In the latter case, the average value of the maximum stress along the CD direction is in fact equal to 70% of the MD value. Another consideration is related to the dispersion of the data which is greater in the MD direction, as the relative distributions are wider and flatter, especially for the T135, US115, and M120 papers. Similar results can be found in the literature [43, 53]. In particular, these last authors highlight that the influence of sample configuration is not significant in measuring Young’s modulus in CD, while it may be significant in MD due to stress concentration.

**Fig. 8:**
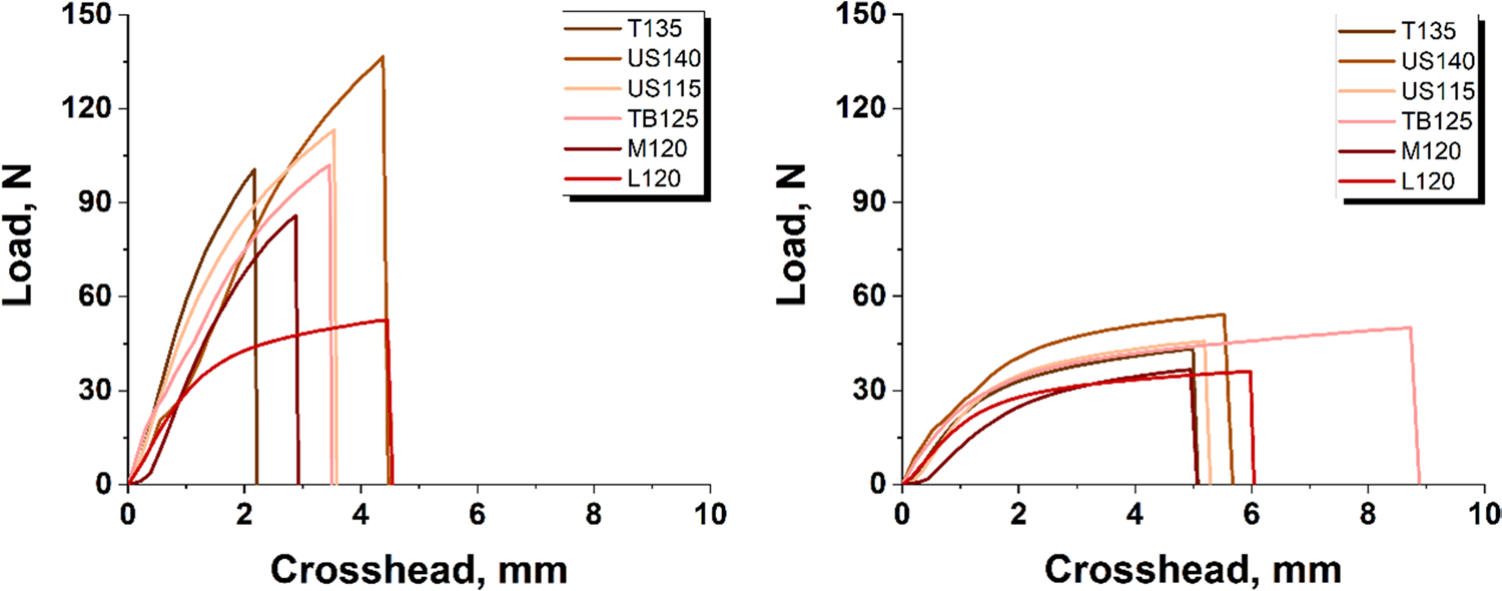
Curve load-crosshead of the paper samples in machine and cross direction

Figure 8 shows the trends of the load–displacement curves of the tensile tested papers in MD (left) and CD (right) directions. The paper tends to be more rigid and resistant in MD, with almost linearly elastic but fragile trends compared to CD, the latter direction in which the same papers show large plastic deformations and ductility as reported in [23]. This is attributable to the orientation of the fibers, but even more important is the fact that, in the production phase, in MD direction the paper is dried with a much greater constraint than in CD [6]. In fact in the direction of the machine, the paper is dried under tension. In the perpendicular direction (CD), the paper strip may shrink to a certain extent [31]. Tables 4, 5, and 6 show the values of the Young (*E*) and shear (*G*) modulus in the different directions and calculated using the formulas (1–4), using the experimental data obtained from the tensile tests. The sample characterized by the higher Young’s modules is US115, while the most resistant paper is US140, able to withstand greater loads than the other types, as shown in the previous Fig. 8.

**Table 4:** Experimental test results for the configuration TUT/323/C

	Test liner 135	Semichemical use 115
*E*_*MD*_ (MPa)	4607.985	4552.469
*E*_*CD*_ (MPa)	1637.488	2034.782
*E*_*TD*_ (MPa)	23.040	22.762
*G*_*xy*_ (MPa)	1063.054	1177.860
*G*_*xz*_ (MPa)	83.782	82.772
*G*_*yz*_ (MPa)	46.785	58.136

**Table 5:** Experimental test results for the configuration TUT/363/B

	Test liner 135	Semichemical use 140
*E*_*MD*_ (MPa)	4607.985	2706.276
*E*_*CD*_ (MPa)	1637.488	1324.252
*E*_*TD*_ (MPa)	23.040	6.621
*G*_*xy*_ (MPa)	1063.054	732.626
*G*_*xz*_ (MPa)	83.782	24.077
*G*_*yz*_ (MPa)	46.785	77.322

**Table 6:** Experimental test results for the configuration TbML/242/E

	Test (white) 125	Medium 120	Liner 120
E_*MD*_ (MPa)	2852.038	3148.311	2114.878
E_*CD*_ (MPa)	1842.939	1339.985	1488.329
*E*_*TD*_ (MPa)	14.260	6.699	10.574
*G*_*xy*_ (MPa)	887.246	794.877	686.599
*G*_*xz*_ (MPa)	51.855	24.363	38.452
*G*_*yz*_ (MPa)	52.655	89.952	42.524

### Edge crush test

**Fig. 9:**
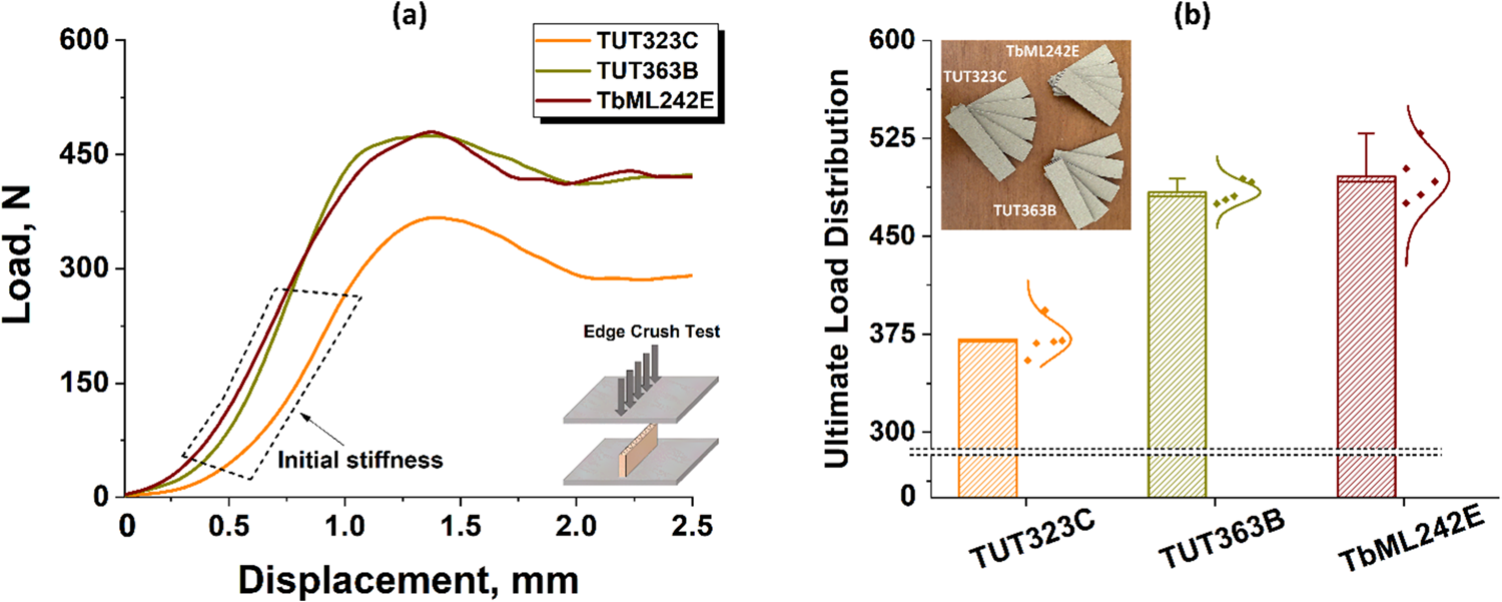
**a** Comparison of ECT curves, **b** ultimate load distribution

The results of the edge crushing tests for the three structure configurations under examination are presented below. The graph on the left (see Fig. 9) shows the trend of the average force–displacement curve for each of the three structure configurations in question, while on the right the degree of dispersion measured on 5 replications is shown. The force–displacement graph shows a region of lower initial stiffness potentially caused by the initial flattening and crushing of the upper and lower shear surface as experimentally found by other authors [44]. The elastic stiffness, which can be determined through the linear part of the experimental curves, cannot be considered equal to the real stiffness because it includes all the effects of the compliance of the crossbar and the imperfections of the sample, visible especially in the initial part of the curve (see Fig. 9). In terms of average maximum load, comparable values are recorded for the two waves B and E, while slightly lower for the C wave. For the latter, the initial stiffness is lower if compared with the other two, which could also be significant of increased sensitivity to damage caused by the sample preparation process [44]. Considering the standardized length of the specimen, the experimental ECT recorded for the three configurations assumes an average value of 4.83 kN/m and 4.96 kN/m for B and E waves, respectively, while for C wave it is 3.71 kN/m. These results confirm what has already been shown by [17], where the high wave C showed higher values of ECT, than the medium wave B. However, the greatest dispersion is observed for the E wave, equal to 4.2% while it is the most contained for the C wave (1.7%). The B wave presents a dispersion in the results equal to 3.7%. In summary, the deviation does not exceed 5%, and, although limited, can be considered usual because due to some heterogeneity of the corrugated cardboard samples, such as local imperfections, lack of parallelism of the edges of the sample, and local detachment of the corrugated layers [21]. Although the specimen is locked on both sides during the test by two parallel PLA plates, to prevent global out-of-plane instability, it is sometimes possible to observe local instability on the upper outer surfaces of the specimens. A slight curvature is observed especially in the outermost area of the sample (Fig. 2c), because it is more sensitive to collapse.

### Box compression test

**Fig. 10:**
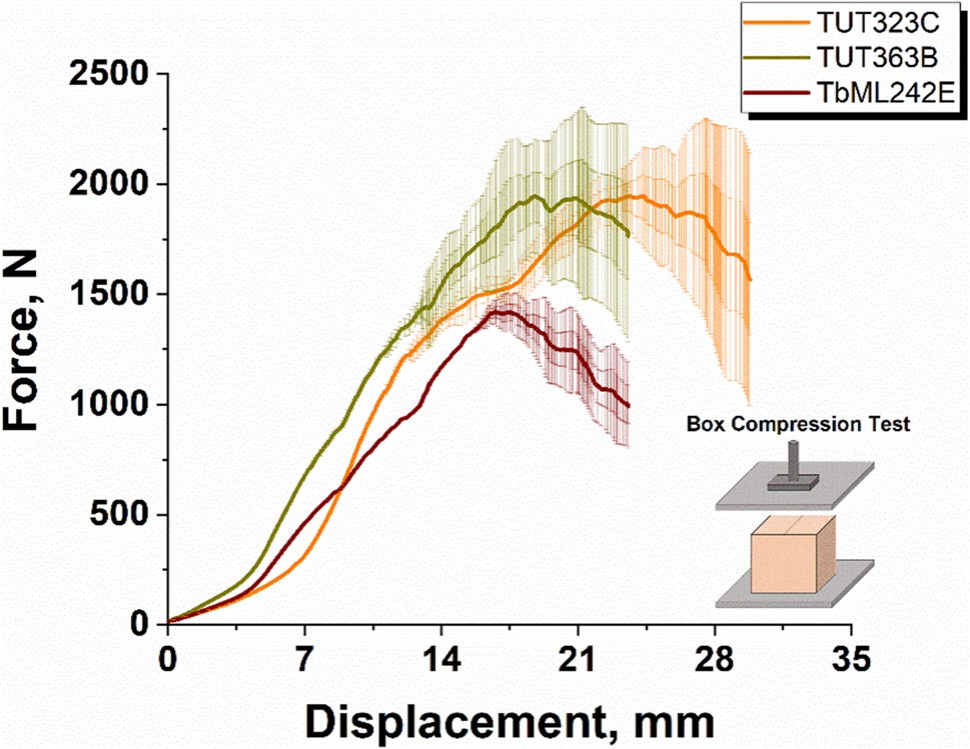
Comparison of BCT curves for American boxes

Figure 10 presents the mean force–displacement curves generated by the compression test for the three chosen configurations, with the relative minimum and maximum dispersions evaluated on five replicates. The two curves, green and yellow, respectively, relating to the high wave C and the medium wave B have provided a high value of the necessary force which is around a value of just over 2000 N. Differences can be highlighted in terms of displacement, as the B wave first reaches the condition of instability. In the case of the E micro-wave, it is possible to observe instead that the condition of instability is reached before both in terms of force and of maximum displacement and before reaching deformation. Thus, the BCT results are between about 2166 and 1469 N. In particular, the maximum load value recorded in the BCT is 2397 N for the C wave with a standard deviation of 223 N (9.26%); for B wave the maximum recorded load is 2119 N with a standard deviation of 266 N (10%); these values are confirmed by [41].

For E wave, on the other hand, the maximum load value recorded is equal to 1518 N and the standard deviation is equal to 928 N (6.10%). In summary, the deviation is between 6 and 10%; however, this dispersion is considered inevitable when working with this type of material [19].

### Homogenization results

Table 7 shows the results of the homogenization process for flutes C, B, and E respectively. As expected, depending on the wave, different thicknesses of the homogenized material are observed. In particular, a thickness of about 5 mm is found for the C wave, just over 3 mm for the B wave and about half of the latter for the E wave. Then in Table 8, the results of the homogenization process of the entire structure for the three configurations studied are reported.

**Table 7:** Homogenization results for the flutes of C, B, and E wave

	C wave	B wave	E wave
*E*_*MD*_ (MPa)	13.3	39.8	87.59
*E*_*CD*_ (MPa)	143.7	229.5	146.8
*G*_*xy*_ (MPa)	10.87	27.25	175
*G*_*yz*_ (MPa)	10.06	10.17	9
*v*_*xy*_	0.018	0.0366	0.1093
Homogenized thickness (mm)	5.017	3.311	1.644

**Table 8:** Homogenization results for the structures with C, B, and E wave

	TUT/323/C	TUT/363/B	TbML/242/E
*E*_*MD*_ (MPa)	348.5	521	500
*E*_*CD*_ (MPa)	202	270.4	451
*E*_*TD*_ (MPa)	1.75	2.6	2.5
*G*_*xy*_ (MPa)	90.1	132	175
*G*_*xz*_ (MPa)	6.5	10	13
*G*_*yz*_ (MPa)	6	8	9
*v*_*xy*_	0.33	0.33	0.32
*v*_*yz*_	0.01	0.01	0.01
*v*_*xz*_	0.01	0.01	0.01

### Simulation results for corrugated cardboard

The entire simulation package is based on the calculation of two subsequent and complementary analyses. In fact, first of all the software determines the solutions of the “static structural” problem, as a function of the boundary conditions just described. Then, the instability analysis called “eigenvalue buckling,” in which the results of the “static structural” section are used as input data, necessary for the definition of the “pre-stress” condition required by the buckling analysis follows. As already mentioned, this is an analysis performed to verify when the instability of the geometry arises under compression loads.

#### Numerical results of edge crush test

**Fig. 11:**
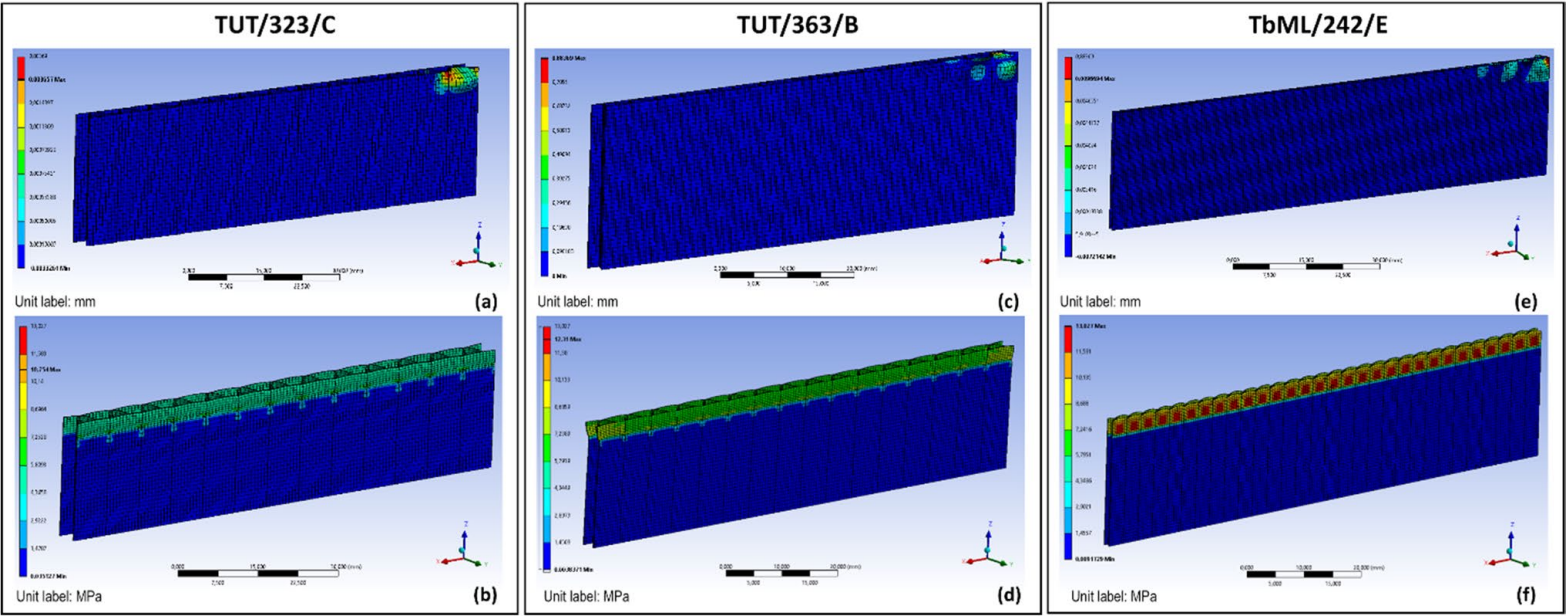
TUT/323/C: **a** eigenvalue buckling, **b** Von Mises stress; TUT/363/B: **c** eigenvalue buckling, **d** Von Mises stress; TbML/242/E: **e** eigenvalue buckling, **f** Von Mises stress

Figure 11 shows the results of the static simulation carried out on the three corrugated cardboard configurations. For each corrugated board configuration, the results are reported in terms of buckling and Von Mises equivalent stresses. Respectively in panels (a), (c), and (e) of Fig. 11, the deformations for C, B, and E waves are reported in correspondence of the load value that induces the first mode of instability in the aforesaid structures. In all three cases, the deformation is concentrated at the upper edge of the specimen and appears as undulation. From a qualitative point of view, the deformation connected to the first mode of instability is consistent in all three case studies. The load value for which this instability condition occurs is different in the three presented corrugated structure configurations. The result is 363 N for the C wave, 471 N for the B wave, and finally, for the E wave configuration, the load that induces instability was estimated to be equal to 498 N. The ECT values, extracted from the FEM simulations, are reported in Table 9, where a comparison with the experimental results is made and the percentage error is calculated. The comparative analysis between the experimental and simulated results guarantees a good reliability of the numerical model that can be considered representative of the real dynamics to which the aforementioned specimens are subjected. In [17], similar results were found in particular for the C wave, where the percentage change between the experimental value and FEM does not exceed 2.5%.

**Table 9:** Experimental results of ECT

Sample	ECT_Exp_ (kN/m)	ECT_FEM_ (kN/m)	Err %
TUT/323/C	3.71	3.63	− 2.15
TUT/363/B	4.83	4.71	− 2.49
TbML/242/E	4.96	4.98	0.4

In panels (b), (d), and (e), instead, the distributions of equivalent stress to which the three configurations are subjected in correspondence of the load value that induces the instability are reported. Von Mises stress distribution can be used to get a qualitative idea of the material behavior. In fact, analyzing qualitatively the distribution of the stresses in the three configurations, it can be observed how this is homogeneous in all three configurations under examination, obviously concentrating in the area of the specimen between the upper edge and the fixed constraint imposed on outer and inner, at 5 mm from the upper edge. The maximum equivalent stress value is reached by E configuration and is approximately 13 MPa. Remember that the E wave is the one that reaches the unstable condition at the highest load value among the three chosen configurations. A very similar value is then obtained from B wave which reaches a maximum value of 12.31 MPa and finally the structure with C wave which is less stressed, in correspondence of the buckling load value. The maximum equivalent stress value is 10.754 MPa. The comparison of the distribution of the equivalent stresses between the tested structures shows how in the case of type-B and type-C wave, the stresses are similarly distributed. The type-E micro-wave shows a higher instability load. The elements subjected to the compressive load are more stressed, showing a concentration of the forces for the layers that are not in contact with the flute.

#### Prediction of double-wave configuration

**Fig. 12:**
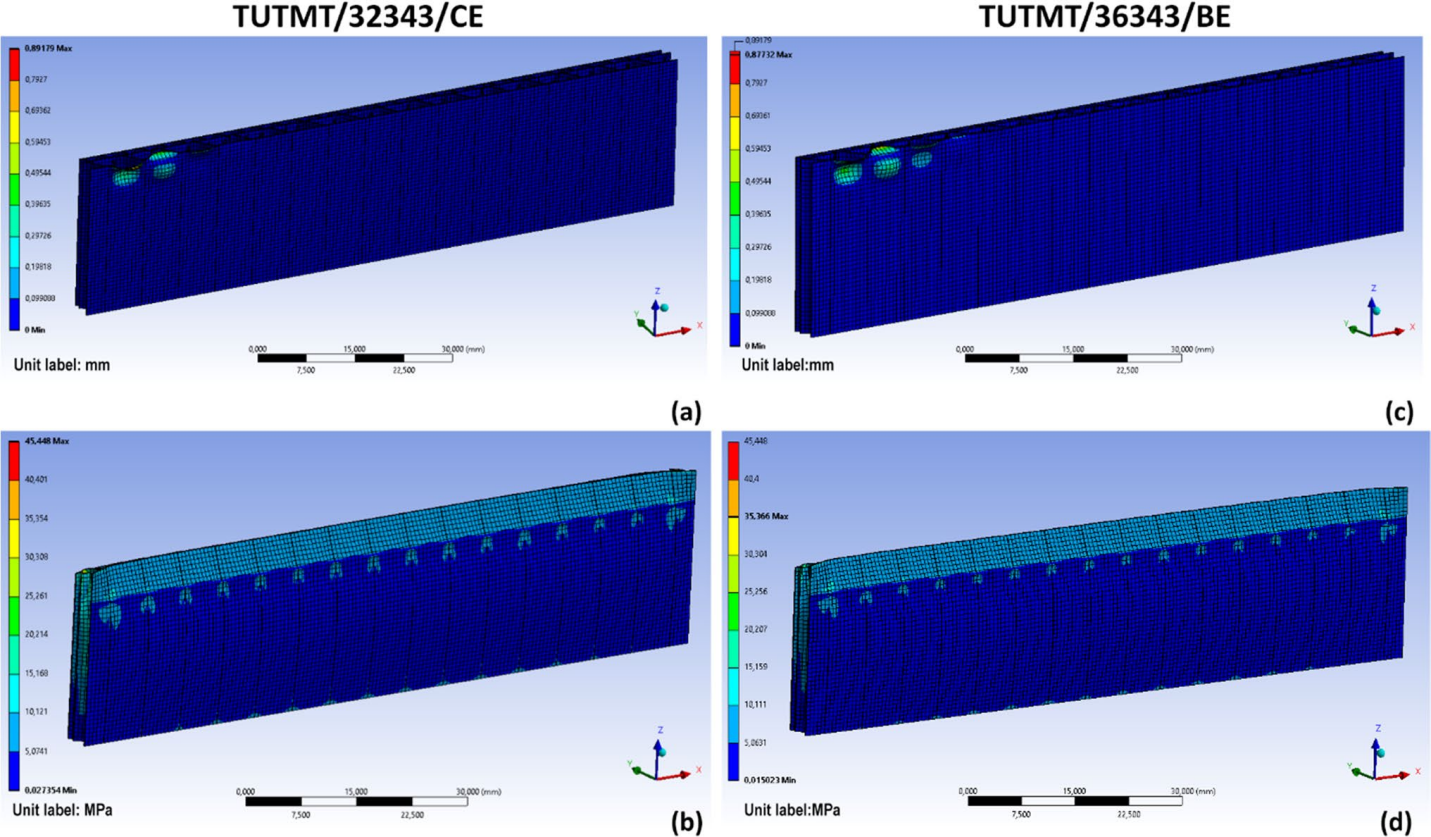
Double-wave configurations: **a**, **c** deformation under load application, **b**, **d** Von Mises stress under load application

The double-wave corrugated combinations considered are TUTMT/36343/BE and TUTMT/32343/CE. The simulation of the behavior of the following structures was considered interesting because of the previous analyses carried out for the single-wave structures. Using the models previously calibrated and validated for single-wave structures, the behavior of these structures was evaluated. The two double-wave structures have in common a constructive characteristic: both configurations are realized by the assembly of a “macro” wave (B or C) and a micro-wave (E). Thus, the boundary conditions that define the test carried out are the same as for single-wave structures. The results of the simulation are shown in Fig. 12. Also in this case, as previously done for single-wave specimens, the results are reported in terms of buckling and equivalent stress according to Von Mises. The sections (a) and (c) show the deformation due to buckling and in both cases it is possible to notice how this is concentrated on the area of the specimen adjacent to the “macro” wave. Qualitatively, the deformation is very similar to that of mono-wave structures. The structures, in terms of critical load, recorded the following values: 777 N for TUTMT/36343/BE and 799.2 N for TUTMT/32343/CE. There is a difference of less than 2% in terms of critical load between the two configurations, as already highlighted in. In terms of equivalent stress, however, always evaluated at the estimated buckling load, the maximum stress value, equal to about 45.45 MPa, is reached by the structure with C-E waves, while the structure with B-E waves reaches at most a value equal to about 35.4 MPa. From a quantitative point of view, therefore, there is a marked difference between the two configurations, while qualitatively they present a distribution of stresses almost similar.

#### Numerical results of box compression test

**Fig. 13:**
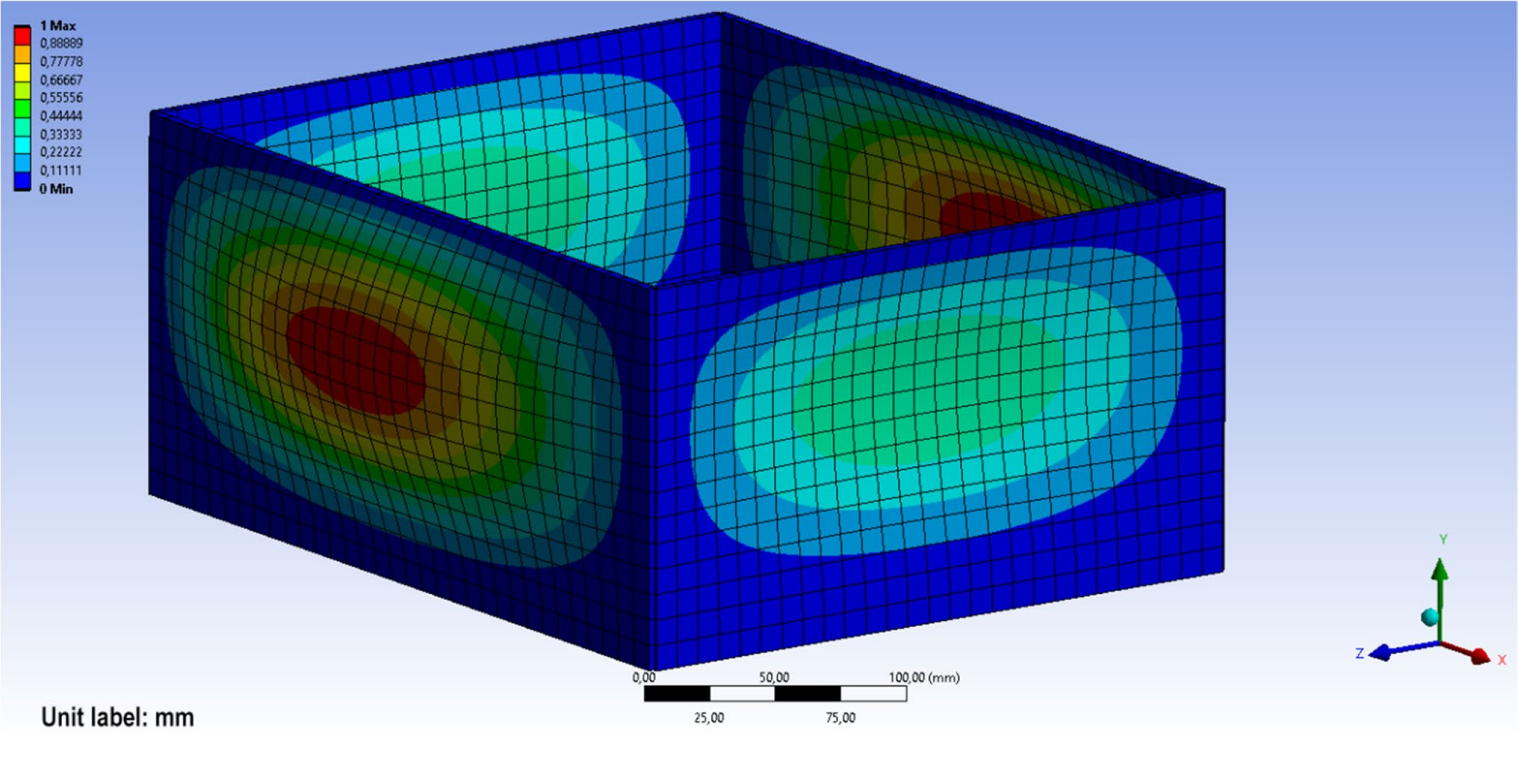
Box configuration: eigenvalue buckling deformation

After the homogenization procedure and consequent implementation of the equivalent mechanical properties, the simulations for the box compression test were iterated, following the conditions described above. In Fig. 13, it is possible to observe the deformation of the box in correspondence of the instability conditions. Only one morphology is reported as the three structures, this time, differ only in terms of equivalent mechanical properties obtained from the homogenization process. Table 10 shows the results offered by the numerical solver, in terms of the maximum load supported by each of the three configurations. Then, in Table 11, the results obtained experimentally and by simulation are compared with the theoretical value. In fact, by applying simplified McKee’s formula, the following values, expressed in kilograms, are derived. The estimate offered by the mathematical formula shows a deviation from the value calculated experimentally or through finite elements, as confirmed in the study of [10]. However, the theoretical value is to be considered reliable only as a first approximation, at least for the case of B and C waves. McKee also found that, because of the approximations made, the simplified formulation gives an underestimate of the ultimate compressive load. Parameters such as the height of the box can greatly influence the buckling phenomenon. In the case of the box with micro-wave E, the most significant gap may be attributable to a construction factor of the structure that is expressed in a greater number of corrugated recurrences per unit length. All three results obtained numerically, thanks to the small percentage gap with experimental results, confirm a good ability of the numerical model to predict the real mechanical-structural behavior of the structures.

**Table 10:** Experimental results of BCT, in terms of buckling load

Sample	*P*_buckl,exp_ (N)	*P*_buckl,FEM_ (N)	Err %
TUT/323/C	2166	2163	− 0.14
TUT/363/B	2094	2090	− 0.2
TbML/242/E	1469	1461	− 0.55

**Table 11:** BCT results comparison with theorical result, in terms of kilograms

Sample	BCT_theorical_ (kg)	BCT_experimental_ (kg)	BCT_FEM_ (kg)
TUT/323/C	214.87	220.79	220.49
TUT/363/B	220.09	213.04	213.04
TbML/242/E	153.55	149.74	148.92

**Fig. 14:**
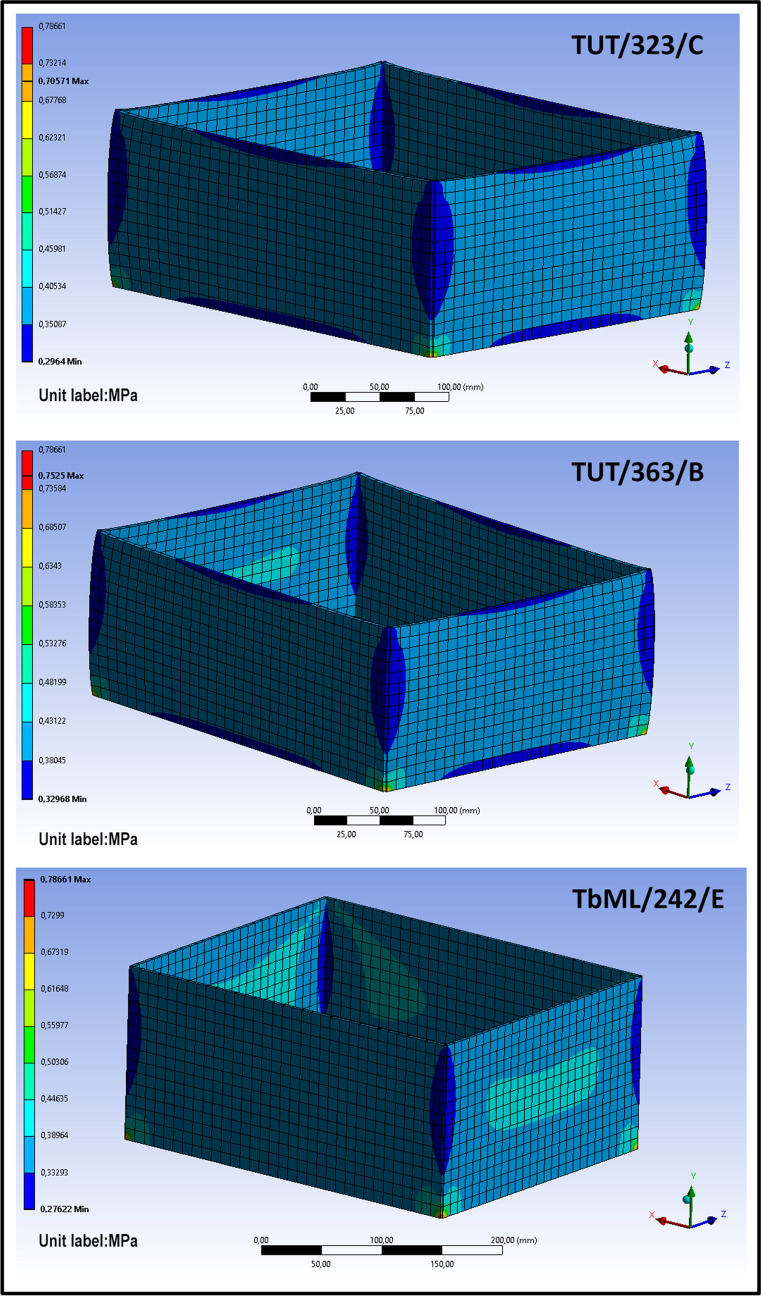
Von Mises stress under load application

Finally, Fig. 14 shows the results for the three configurations, in terms of equivalent stresses, calculated at the Buckling load identified for each of them. A qualitative analysis of the stress state of the structure allows to identify the areas that are most affected by the application of the external load. An overview of the structural behavior of the specific configuration can be achieved. As expected, at the relative buckling load, the structure that reaches higher equivalent stress values is the configuration with E wave. At the corners of the box, this structure achieves a maximum value equal to 0.78 MPa. For the configuration with B wave, the value is 0.75 MPa, and finally for the structure with C wave the value is 0.7 MPa. However, wanting to make a hypothesis about the resistance of the three structures, it is good to consider not so much the peak values achieved, but its distribution along the entire geometry. Figure 14 shows that, among the three configurations, the type-E wave is the most stressed, while the type-B and type-C waves feature lower stress, as they develop less stresses along the entire geometry. In this regard, (Han and Park, 2007) developed a model to determine the mechanical behavior of corrugated boxes, where the maximum stresses were found beside the corners of the box, while the walls of the box are less stressed, being this in strict agreement with the results of the present work. Indeed, [29] also underlined how the response of the box to the compressive load depends mainly on its geometric conformation, as it is herein stated as well.

## Conclusions

The present work deals with a comparative evaluation of the mechanical response of corrugated cardboards designed with different wave configurations. In particular, the focus was paid on wave configurations that allow to reduce the weight of the box, the fabrication cost, and the related environmental impact. More specifically, light single-wave solutions with the micro-wave (type E) and cost saving double-wave solutions obtained by pairing the lighter micro-wave (type E) to a standard wave (medium wave, type B, or high wave, type C) were comparatively evaluated to standard high-weight solutions.

Tensile tests, edge compression tests (ECT), and BCT were performed on the different types of wave configurations. The same configurations investigated by the aforementioned experimental tests were also studied by simulating their response by finite element method (FEM). The results of the FEM models are in good agreement with the experimental results. Maximum error recorded is 2.5% for ECT and 0.55% for BCT. The FEM model of ECT was also used for evaluating the mechanical performance obtained on dual-wave structures, that means by pairing the lighter micro-wave (type E) to a standard wave (medium wave, type B, or high wave, type C), respectively.

Based on the experimental and numerical evidences, the micro-wave (type E) is found to be only slightly less effective than the standard medium waves of type B and type C, indicating the high potential of the type E wave in multiple applications. Accordingly, involving the micro-wave (type E) in the manufacturing of corrugated board allows reducing the ecological footprint of the resulting boxes. Furthermore, the micro-wave (type E) leads to both a reduction in the fabrication cost of the corrugated cardboard and a decrease in the final weight of the packaging.

In conclusion, type-E micro-wave can be considered an excellent replacement of the heavier type-B and type-C waves. In addition, pairing of the micro-wave type-E with traditional waves (both type B and type C) is of utmost interest, as it can combine high performance with rationale design of the packaging and high savings, even for high challenging applications.
